# International Family Nursing Association: toolkit of resources for caring for refugee/migrating families

**DOI:** 10.1590/1980-220X-REEUSP-2022-0446en

**Published:** 2023-06-23

**Authors:** Fernanda Lise, Donna Marvicsin, Debbie Sheppard LeMoine, Norma Krumwiede, Yuuko Mabrey Johnson, Maria do Céu Aguiar Barbieri Figueiredo

**Affiliations:** 1 Universidade Federal de Pelotas, Pelotas, RS, Brazil.; 2 University of Michigan, Ann Arbor, USA.; 3 University of Windsor, Windsor, Canada.; 4 Minnesota State University-Mankato, Mankato, USA.; 5 University of California, Sacramento, USA.; 6 Universidad de Huelva, Departamento de Enfermagem, Huelva, Espanha.

**Keywords:** Family Nursin, Emigration and Immigratio, Refugees, Enfermería de la Familia, Emigración e Inmigración, Refugiados, Enfermagem Familiar, Emigração e Imigração, Refugiados

## Abstract

**Objective:**

To report the experience of the International Family Nursing Association (IFNA) Practice Committee on developing a Toolkit of resources to care for refugee/migrating families as a response to the global migration and refugee crisis.

**Method:**

Qualitative and descriptive study, experience report, which describes the development of a toolkit of resources for caring for refugee/migrating families.

**Results:**

The development of this Toolkit of resources to care for refugee/migrating families is supported by current literature related to family-centered evaluation and intervention, culturally sensitive practice based on family strengths; statements of positioning on immigrant and refugee families; and nursing and health organizations that addressed the health of the refugee family.

**Conclusions:**

The dissemination of the resources available in the Toolkit can support nursing practices, drive qualified approaches to assessments and interventions, capable of promoting family resilience as they adapt, providing well-being, and leading to the healing of traumas and adversities experienced by families in the process of migration or refuge.

## RESUMO

**Objetivo:**

Relatar a experiência do Comitê de Práticas da Associação Internacional de Enfermagem da Família em relação ao desenvolvimento de uma ferramenta com recursos para os cuidados a famílias de migrantes ou refugiados como resposta à crise mundial de migrantes e refugiados.

**Métodos:**

Estudo qualitativo e descritivo. Relato de experiência que descreveu o desenvolvimento de uma ferramenta com recursos para os cuidados a famílias de migrantes ou refugiados.

**Resultados:**

O desenvolvimento dessa ferramenta foi embasado na literatura atual relacionada a avaliação e intervenção centrada na família, uma prática culturalmente sensível baseada nos pontos fortes familiares; em declarações que indicavam o posicionamento sobre as famílias de migrantes ou refugiados; e em organizações de enfermagem e saúde que abordaram a saúde da família refugiada.

**Conclusões:**

A disseminação dos recursos disponíveis na ferramenta pode auxiliar nas práticas de enfermagem, estimular o uso de abordagens adequadas em avaliações e intervenções e promover a resiliência das famílias enquanto elas passam pelo período de adaptação, proporcionando, assim, bem-estar e levando à cura de traumas e adversidades enfrentados pelas famílias no processo de migrar ou refugiar-se.

## RESUMEN

**Objetivo:**

Informar la experiencia del Comité de Prácticas de la Asociación Internacional de Enfermería de Familia respecto del desarrollo de una herramienta con recursos para atención de familias de migrantes o refugiados como respuesta a la crisis mundial de ambos grupos.

**Métodos:**

Estudio cualitativo y descriptivo. Informe de experiencia describiendo el desarrollo de una herramienta con recursos de atención a familias de migrantes o refugiados.

**Resultados:**

El desarrollo de la herramienta se fundamentó en literatura actual relativa a la evaluación e intervención centrada en la familia, práctica culturalmente sensible basada en los puntos fuertes familiares; en declaraciones indicando posición sobre las familias de migrantes o refugiados; y en organizaciones de enfermería y sanitarias que abordaron la salud de las familias refugiadas.

**Conclusiones:**

La difusión de los recursos disponibles en la herramienta puede ayudar en las prácticas de enfermería, impulsar el uso de abordajes adecuados en evaluaciones e intervenciones, y promover la resiliencia en las familias mientras enfrentan el período de adaptación, brindando bienestar y apuntando a sanar traumas y adversidades enfrentadas por las familias en su proceso de migración o refugio.

## INTRODUCTION

The COVID-19 pandemic and conflicts and wars aggravated the health condition of migrant and refugee families around the world and intensified the need for theoretical, culturally sensitive, supported evidence and practices that promote the necessary support for nurses to care for the health of families in migration or refuge situation.

Migration can be a voluntary or involuntary act, for example, someone crossing a border in search of better living conditions, to study or work^(1)^, thus the population migration flow can be motivated by social, economic, cultural, religious, and environmental factors^(2)^. The refugee situation is described as a condition in which people are outside their home countries due to fears of persecution, conflict, violence, or other circumstances that seriously disrupt public order and require “international protection.” Thus, the situations faced are often so dangerous and intolerable that these people decide to cross national borders to seek security in other countries^(1)^.

It is estimated that 82.4 million people worldwide were forcibly displaced by the end of 2020, the highest level ever recorded. About 26.4 million are refugees, and about half are under 18 years old^(3)^. In addition, 258 million people live outside their country of birth, which represents an increase of 49% since 2000^(4)^. Of these 258 million, about 21.3 million are refugees, 40.8 million are internally displaced persons, and 3.2 million are asylum seekers. However, only a small percentage of this population will reach a developed “resettlement” country; therefore, most will eventually settle into “host” countries near their home country. About a third live in camps and the others settle in urban areas^(3)^. Currently, 4.6 million refugees have fled Ukraine since February 24th, 2022, and more than 7 million people are internally displaced in Ukraine. Thus, international migration has become a critical concern for the implementation of the 2030 Agenda for Sustainable Development^(4)^. This study aimed to share the experience report of the development of a resource toolkit to care for refugee/migrating families as a response to the global migration and refugee crisis of the International Family Nursing Association (IFNA) Practice Committee.

## METHODS

### Study Design

This is a qualitative, descriptive study that presents an experience report of the International Family Disease Association (IFNA) Practice Committee, involving the process of developing a toolkit of resources to care for refugee/migrant families as a response to the global migration crisis.

### Setting

The IFNA Practice Committee is made up of family health nurses from Australia, Brazil, Canada, Japan, New Zealand, Portugal, South Africa, Spain, United States, and Taiwan. This committee has an agenda of activities and meets monthly in remote meetings to discuss, plan, and make available the best nursing approaches to families. This committee is tasked with transforming family health by serving as a unifying force and voice for family sickness globally, as well as sharing knowledge, practices, and skills to enhance and nurture family sickness practice; also providing leadership in family sickness through education, research, socialization, and collegiate exchange in all aspects of family sickness. The toolkit development process is supported by current literature related to family-centered assessment and intervention, a culturally sensitive practice based on family strengths; statements of position on immigrant and refugee families; and health and sick organizations that will address the health of the refugee family.

### Ethical Aspects

This study reflects the opinions of the authors and the guidelines contained in the resource toolkit for caring for refugee/migrating families are supported by scientific studies, statements from government organizations, councils, and sick associations concerned with the health of families of migrating and refugee individuals. As it did not require any form of data collection, there was no need for submission to an ethics committee.

## RESULTS

**Figure 1.**
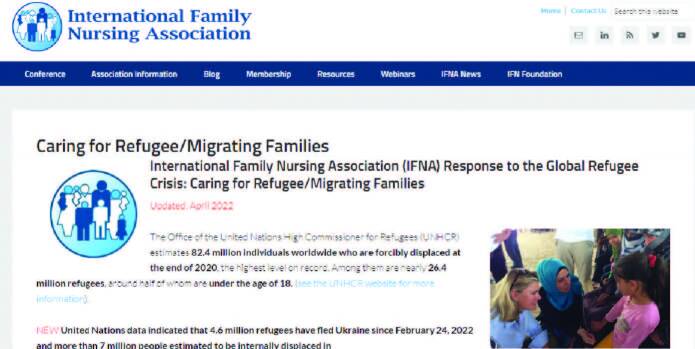
Resource Toolkit for Caring for Refugee/Migrating Families^(5)^.

Source: International Family Nursing Association^(5)^.

This is an experience report of the development of a toolkit resource for the care of migrating and refugee families by the IFNA Practice Committee. A subcommittee was created in the Practice Committee to systematically identify internationally produced documents (scientific articles, practice guidelines, online resources) and best practices and disseminate this information on the IFNA website. This committee brings together specialists in family nursing, in monthly meetings to discuss the best practices in family care, family nursing skills, and political issues that affect the provision of family nursing care, as we understand that efforts to reduce the suffering of families need to be made by the whole society. In this sense, family nurses recognize the need to be instrumentalized in the development of culturally sensitive care, based on theoretical models and scientific and practice evidence. In the process of developing the Resource Toolkit for Caring for Refugee/Migrating Families, printed and media resources were identified by members of the Practice Committee, and selected and made available online. Once it was determined that the information in the resource was relevant to promoting, maintaining, and restoring the health of immigrant families as to promoting care for migrating and/or refugee families, the resource was added to the IFNA website^(5)^, designed to support families and health professionals in the care of migrating individuals and/or refugees (Figure 1).

Given this emerging theme for nursing and the need for an immediate response to meet the global migration and refugee crisis, the IFNA developed a toolkit of resources to care for migrating/refugee families^(5)^. This toolkit is supported by 1) Current Literature: Family-focused Assessment and Intervention of Refugee Families; 2) statements of positioning on immigrant and refugee families; and 3) information from nursing and health organizations that addressed the health of the refugee family.

### IFNA Recommendations for Practices with Migrant and Refugee Families

Based on the evidence of the studies, we propose some recommendations to promote the health of migrating or refugee families based on studies developed with families in different cultures:

– Provide high-quality health care to all individuals who make up migrating or refugee families regardless of age, gender, language, or ethnic origin.
– Culturally sensitive health care is based on cultural beliefs, practices, and values, which facilitate access for migrating or refugee individuals and families to the use of health services.
– Establish appropriate verbal and non-verbal communication for the transmission of clear messages and request feedback from migrating or refugee individuals and families.
– Promote integration into the new environment with integration to the community, school, church, work, language, and culture in the new community, linking migrating or refugee individuals and families to health groups.
– Reduce the socio-cultural distance between migrating or refugee professionals, individuals, and families.
– Promote measures to approach home visits and search for migrating or refugee individuals and families, favoring the relationship of trust and empathy.
– Promote dialogue on the perception of the family in the face of differences in cultural patterns and behaviors comparing their country of origin and the new country.
– Increase the capacity to adapt to the changes to reduce the bio-psychosocial risks of migrating or refugee individuals and families.
– Promote commitment of nurses’ associations and councils to increase efforts to make nursing services compulsory in schools to facilitate access for migrating or refugee individuals and families to health care.
– Recognize that migrating or refugee families face a complex experience due to the nature of migration and the acculturation process.
– Keeping families together should be a priority, since family separation, and even the threat of separation harms children.
– Train family nurses to perform the screening of emotional suffering for migrating and refugee individuals and families.
– Encourage the practice of physical activities such as collective sports that contribute to the socialization and learning of the new language.

## DISCUSSION

### Current Literature: Family-Focused Assessment and Intervention of Refugee Families

Providing access to updated, evidence-based and practice- based literature is a strategy to support the instrumentalization of nurses in their practices with families^(5)^. Research developed in different countries has contributed to the knowledge about the complex situations of life of migrating individuals and refugees^(6)^, however, information on their experiences in health systems in their host countries shows that the need to improve the quality of health care is urgent^(7)^.

Using a theoretical model of care in the approach to migrating/refugee families is recommended^(8,9)^. As an intervention model, the Samarasinghe refugee family is based on a comprehensive understanding of the impact of the transition on family health in families of involuntary migrants^(10)^. The development of this model was inspired by the Calgary family-centered care model to be a tool for nurses in the primary health care field, in the context of evaluation and intervention in the family adaptation of families of involuntary migrants in cultural transition, based on the following objectives: (1) manage the acculturation process, (ii) integrate the family into society; (iii) achieve stable family relationships.

In addition to the theoretical models used by nurses in the approach to migrant or refugee families, the actions developed by nurses in schools can be important resources with a strong impact on the construction of bonds, allowing access to health problems^(11)^. Thus, the availability of school nurses as health professionals provides important resources for children and families, and serves as a bridge to connect families to health services^(12)^. In addition, providing appropriate nursing therapies can promote a healthy transition in the general context of migration from the acculturation process, physical, biological, economic, political, social and psychological changes that occur in the learning of a new lifestyle^(10)^.

### Position Statements About Refugees/Migrating Families

As family nurses, our attitudes toward the global refugee crisis are intrinsically linked to our beliefs about the central importance of the family, our ethics of family rights to health and well-being, and our role in promoting family health. Family nurses have always been in a unique position to help families who are experiencing physical, psychological, relational, and/or spiritual difficulties of forced migration and displacement due to armed conflicts, violence, persecution, poverty, and disasters^(5)^.

Other international organizations concerned with the lives of migrants and refugees have declared their position on the host policies adopted by countries, contrary to the separation of children and their parents, as it can trigger a high level of toxic stress and psychological trauma. Thus, they highlight the importance of maintaining the union of members to reduce stress and improve well-being^(13,14)^.

Keeping families together should be a priority, as family separation, and the threat of separation harm children (National Council on Family Relations). In addition, parents suffer from the constant threat of separation from their children and feel discouraged by the way their undocumented status affects family processes^(15)^. Women/mothers suffer from the distance of the children left behind, when they migrate without their children or continue to be responsible for the care of their children, while their husbands are in another country^(16)^.

Families of refugees and migrants need to be empowered, and cooperation between countries should strengthen the resilience of refugee communities, particularly in developing countries. Migration and orderly, safe, regular, and responsible mobility of people through the implementation of planned and well-managed migration policies should be facilitated^(4)^.

Addressing the health needs and suffering of families (especially children) who have experienced adverse forced migration experiences is one of the first steps to providing quality family nursing care. Promoting the resilience of families as they adjust to acculturation and resettlement, as well as healing adversity and trauma, requires that family nurses be highly qualified. Family nurses bring an acute awareness of the complex interactions between the individual, the family, and the wider social and cultural contexts in which refugees resettle. Wherever families are in need, family nurses will be there to offer compassion, curiosity, and healing while advocating policies and access to resources that will transform family health around the world^(5)^.

### Nursing and Health Care Organizations: Documents and Websites About Refugee/Migrating Family Health

The documents made available by nursing and health entities on the health of migrating/refugee families present updated information with policy and care guidelines. Since efforts to reduce the suffering of families need to be made by the whole society, family nurses recognize the need to be instrumentalized in the development of culturally sensitive care based on theoretical models^(17,18,19,20,21,22,23)^.

## STUDY LIMITATIONS

The limitations of this study are related to sociocultural aspects because it is very difficult to develop a unique guide or manual that fits the reality of all families of migrating individuals or refugees, given the great cultural diversity of communities, even for those who come from the same country. Therefore, the guidelines contained in this study are considered generic and pertinent to be used in different scenarios and cultures.

## FINAL CONSIDERATIONS

The toolkit for caring for refugee/migrating families developed by the International Family Nursing Association (IFNA) is a response to the global migration and refugee crisis, and is supported by current literature related to family-centered assessment and intervention, culturally sensitive and based on strengths; position statements about immigrant and refugee families; and information from nursing and health organizations that addressed the health of the refugee family in practice with individuals and families, emphasizing the important role that nursing plays in practice with migrating or refugee families at a global level. This is especially important in welcoming families, reducing fear, and favoring the construction and strengthening of links with the support network made up of social organizations such as schools, churches, and work so that the acculturation process occurs without trauma. Therefore, it is considered essential that nurses develop skills to care with empathy, respect for cultural differences, and advocate for families.
